# The Genia Event and Protein Coreference tasks of the BioNLP Shared Task 2011

**DOI:** 10.1186/1471-2105-13-S11-S1

**Published:** 2012-06-26

**Authors:** Jin-Dong Kim, Ngan Nguyen, Yue Wang, Jun'ichi Tsujii, Toshihisa Takagi, Akinori Yonezawa

**Affiliations:** 1 Database Center for Life Science, Research Organization of Information and Science, 2-11-16 Yayoi, Bunkyo-ku, Tokyo, Japan; 2 Department of Information science, University of Tokyo, 7-3-1 Hongo, Bunkyo-ku, Tokyo, Japan; 3 Microsoft Research Asia, 5 Dan Ling Street, Haidian District, Beijing, China; 4 Department of Computational Biology, University of Tokyo, 5-1-5 Kashiwa-no-ha, Kashiwa, Chiba, Japan

## Abstract

**Background:**

The Genia task, when it was introduced in 2009, was the first community-wide effort to address a fine-grained, structural information extraction from biomedical literature. Arranged for the second time as one of the main tasks of BioNLP Shared Task 2011, it aimed to measure the progress of the community since 2009, and to evaluate generalization of the technology to full text papers. The Protein Coreference task was arranged as one of the supporting tasks, motivated from one of the lessons of the 2009 task that the abundance of coreference structures in natural language text hinders further improvement with the Genia task.

**Results:**

The Genia task received final submissions from 15 teams. The results show that the community has made a significant progress, marking 74% of the best F-score in extracting bio-molecular events of simple structure, e.g., gene expressions, and 45% ~ 48% in extracting those of complex structure, e.g., regulations. The Protein Coreference task received 6 final submissions. The results show that the coreference resolution performance in biomedical domain is lagging behind that in newswire domain, cf. 50% vs. 66% in MUC score. Particularly, in terms of protein coreference resolution the best system achieved 34% in F-score.

**Conclusions:**

Detailed analysis performed on the results improves our insight into the problem and suggests the directions for further improvements.

## Background

The BioNLP Shared Task (BioNLP-ST, hereafter) is a series of efforts to promote a community-wide collaboration towards fine-grained information extraction (IE) in biomedical domain. The first event, BioNLP-ST 2009, introducing a bio-molecular event (bio-event) extraction task, attracted a wide attention, with 24 teams submitting final results [1].

To establish a community effort, the organizers provided the task definition, benchmark data, and evaluations, and the participants competed in developing systems to perform the task. Meanwhile, participants and organizers communicated to develop a better setup of evaluation. Some participants provided their tools and resources for others, making it a collaborative competition.

The final results showed that the automatic extraction of simple events - those with unary arguments, e.g., gene expression - could be achieved at the performance level of 70% in F-score, but the extraction of complex events, e.g., binding and regulation, was a lot more challenging, having achieved 40% of performance level.

After BioNLP-ST 2009, all the resources from the event were released to the public, to encourage continuous efforts for further advancement, and the online evaluation service has been kept open to provide reliable evaluation. Since then, several improvements have been reported [2-6]. For example, Miwa et al. [2] reported a significant improvement with binding events, achieving 50% of performance level.

The task introduced in BioNLP-ST 2009 was renamed to *Genia event (GE) task*, and was hosted again in BioNLP-ST 2011, which also hosted four other main tasks and three supporting tasks [7]. As the sole task that was repeated in the two events, the GE task took the role of connecting the results of the 2009 event to the other main tasks of 2011. The GE task in 2011 received final submissions from 15 teams. The results show that the community made a significant progress with the task, and that the technology can be generalized to full papers at moderate cost of performance.

It is one of the lessons from BioNLP-ST that coreference structures in biomedical text substantially hinder the progress of fine-grained IE [1]. To address the problem, the Protein Coreference (CO) task was arranged as one of the supporting tasks of BioNLP-ST 2011. While the task itself is not an IE task, it is expected to be a useful component in performing the main IE tasks more effectively. To establish a stable evaluation and to observe the effect of the results of the task to the main IE tasks, the CO task particularly focused on finding anaphoric protein references. After 7 weeks of system development phase, six teams submitted their final results. According to our primary evaluation criteria, the best system is evaluated to find 22.18% of anaphoric protein references at a precision of 73.26%.

This paper presents the results of BioNLP-ST 2011, extending the GE and CO task overview papers [8,9] in the BioNLP-ST 2011 workshop proceedings. Particularly, the paper focuses on providing more data and analyses to support the improvement and generalization achieved with the GE task, and also to show the problems of current approaches of CO with possible future directions.

## Results and discussions

The results of BioNLP-ST 2011 are summarized to the task definition, resources and results.

### Task definitions

#### GE task

The GE task follows the task definition of BioNLP-ST 2009, which is briefly described in this section. For more detail, please refer to [1,10].

Table 1 shows the event types to be addressed in the task. For each event type, the primary and secondary arguments to be extracted with an event are defined. For example, a *Phosphorylation *event is primarily extracted with the protein to be phosphorylated. As secondary information, the specific site to be phosphorylated needs to be extracted when expressed in text.

**Table 1:** Event types and their arguments for the GE task.

Event Type	Primary Argument	Secondary Argument
Gene_expression	Theme(Protein)	
Transcription	Theme(Protein)	
Protein_catabolism	Theme(Protein)	
Phosphorylation	Theme(Protein)	Site(Entity)
Localization	Theme(Protein)	AtLoc(Entity), ToLoc(Entity)

Binding	Theme(Protein)+	Site(Entity)+

Regulation	Theme(Protein/Event), Cause(Protein/Event)	Site(Entity), CSite(Entity)
Positive_regulation	Theme(Protein/Event), Cause(Protein/Event)	Site(Entity), CSite(Entity)
Negative_regulation	Theme(Protein/Event), Cause(Protein/Event)	Site(Entity), CSite(Entity)

The type of each filler entity is specified in parenthesis. Arguments that may be filled more than once per event are marked with "+".

From a computational point of view, the event types represent different levels of complexity. When only primary arguments are considered, the first five event types in Table 1 are classified as *simple event types*, requiring only unary arguments. The *Binding *and *Regulation *types are more complex: *Binding *requires detection of an arbitrary number of arguments, and *Regulation *requires detection of recursive event structure.

Based on the definition of event types, the entire task is divided to three sub-tasks addressing event extraction at different levels of specificity:

**Task 1. Core event extraction **addresses the extraction of typed events together with their primary arguments.

**Task 2. Event enrichment **addresses the extraction of secondary arguments that further specify the events extracted in Task 1.

**Task 3. Negation/speculation detection **addresses the detection of negations and speculations over the extracted events.

Task 1 serves as the backbone of the GE task and is mandatory for all participants, while the other two are optional.

**Figure 1:**
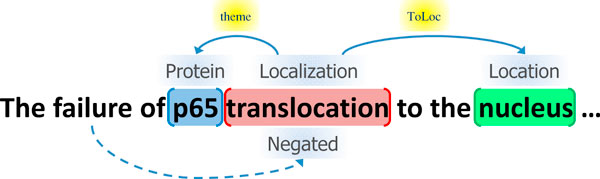
**Event annotation example**.

Figure 1 shows an example of event annotation. The event encoded in the text is represented in a standoff-style annotation as follows:

T1Protein 15 18

T2 Localization 19 32

T3 Entity 40 46

E1 Localization:T2 Theme:T1 ToLoc:T3

M1 Negation E1

The annotation T1 identifies the entity referred to by the string between the character offsets, *15 *and *18 *(*p65*) to be a *Protein*. T2 identifies the string, *translocation*, to refer to a *Localization *event. Entities other than proteins or event type references are classified into a default class *Entity*, as in T3. E1 then represents the event type and the arguments, as defined in Table 1. Note that for Task 1, the entity, T3, does not need to be identified, and the event, E1, may be identified without specification of the secondary argument, ToLoc:T3:

E1' Localization:T2 Theme:T1

Finding the full representation of E1 is the goal of Task 2. In the example, the localization event, E1, is negated as expressed in *the failure of*. Finding the negation, M1, is the goal of Task 3.

#### CO task

**Figure 2:**
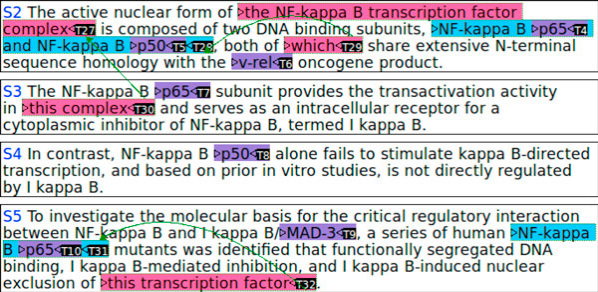
**Protein coreference annotation**.

The CO task is newly defined in BioNLP-ST 2011. Figure 2 shows an example text that is segmented into four sentences, S2 - S5, where anaphoric coreferences are illustrated with colored extends and arrows. In the figure, protein names are highlighted in purple, T4 - T10, and anaphoric protein references, e.g., pronouns and definite noun phrases, are highlighted in red, T27, T29, T30, T32, of which the antecedents are indicated by arrows if found in the text. In the example, the definite noun phrase (NP), *this transcription factor *(T32), is a coreference to *p65 *(T10). Without knowing the coreference structure, it becomes hard to capture the information written in the phrase, *nuclear exclusion of this transcription factor*, which is *localization of p65 (out of nucleus) *according to the framework of BioNLP-ST.

A standard approach would include a step to find candidate anaphoric expressions that may refer to proteins. In this task, pronouns, e.g., *it *or *they*, and definite NPs that may refer to proteins, e.g., *the transcription factor *or *the inhibitor *are regarded as candidates of anaphoric protein references. This step corresponds to the *markable detection *and the *anaphoricity determination *steps in the jargon of MUC [11]. The next step would be to find the antecedents of the anaphoric expressions. This step corresponds to the *anaphora resolution *step.

The protein annotation to the example text in Figure 2 is as follows:

T4 Protein 275 278 p65

T5 Protein 294 297 p50

T6 Protein 367 372 v-rel

T7 Protein 406 409 p65

T8 Protein 597 600 p50

T9 Protein 843 848 MAD-3

T10 Protein 879 882 p65

The first line indicates that there is a *protein *reference, *T4*, in the span that begins at *275'th *and ends before *278'th character*, of which the text is *p65*.

The coreference annotation is made by three types of annotations. The first type is the annotations for anaphoric protein references. For example, those in red in Figure 2 are anaphoric protein references:

T27 Exp 179 222 the N. . 215 222 complex

T29 Exp 307 312 which

T30 Exp 459 471 this . . 464 471 complex

T32 Exp 1022 1047 this . . 1027 1047 tra. .

The first line indicates that there is an anaphoric protein reference in the specified span, of which the text is *the NF-kappa B transcription factor complex *(here truncated to five characters due to limit of space), and that its minimal expression is *complex*. The second type is the annotations for the noun phrases that are antecedents of the anaphoric references. For example, *T28 *and *T31 *(highlighted in blue) are antecedents of *T29 *and *T32*, respectively:

T28 Exp 264 297 NF-ka. .

T31 Exp 868 882 NF-ka. .

The last type is the annotations to link the anaphoric expressions to their their antecedents:

R1 Coref Ana:T29 Ant:T28 [T5, T4]

R2 Coref Ana:T30 Ant:T27

R3 Coref Ana:T32 Ant:T31 [T10]

Note that due to limit of space, argument names are abbreviated, e.g., "Ana" for "Anaphora", and "Ant" for "Antecedent". The first line indicates that there is a *coreference *relation, *R1*, of which the *anaphor *is *T29 *and the *antecedent *is *T28*, and that the *antecedent *contains two protein names, *T5 *and *T4*.

Note that, sometimes, an anaphoric expression, e.g., *which *(T29), is connected to more than one protein names, e.g., *p65 *(T4) and *p50 *(T5). Sometimes, coreference structures do not involve any specific protein name, e.g., T30 and T27. In order to establish a stable evaluation, our primary evaluation will focus only on coreference structures that involve specific protein names, e.g., T29 and T28, and T32 and T31. Among the three, only two, R1 and R3, involve specific protein references, T4 and T5, and T10. Thus, finding of R2 will be ignored in the primary evaluation. However, those not involving specific protein references are also provided in the training data to help system development, and will be considered in the secondary evaluation mode.

### Task resources

In order to guide and promote the development of the systems to perform the GE and CO tasks, benchmark data sets were developed and provided to the participants

The benchmark data for the GE task was initially prepared for the first BioNLP Shared Task in 2009. At that time, the data included only titles and abstracts of papers from Medline. For BioNLP-ST 2011, full papers have been added, and the benchmark data now consist of two collections. The *abstract collection *is the same as the data for BioNLP-ST 2009, and is meant to be used to measure the progress of the community. The *full text collection *contains full papers which are newly annotated, and is meant to be used to measure the generalization of the technology to full papers. The whole data sets include annotations for events as defined in Table 1. The abstract collection also include annotations for coreferences, becoming the benchmark data set for the CO task.

The whole data set is divided into three sub-sets for the purpose of training, tuning and testing. The training and tuning sets are provided to the shared task participants, with the full annotations. However, with the test set, only the protein annotations are provided, and the participants are expected to produce the remaining annotations. Table 2 shows basic statistics of the annotations in the benchmark data sets. The number of words shows there are much more training data from abstracts than from full papers. The number of annotated coreferences are shown in classification, indicating that relative pronouns, pronouns, and definite noun phrases are the three major types of anaphora expression in the data set. The number of event annotations shows that *Gene*_*expression*, *Binding *and *Regulation *(including its subtypes) are the most frequent event types in the data sets.

**Table 2:** Statistics of the benchmark data sets for the GE and CO tasks.

	Training		Tuning		Test	
Item	**Abs**.	Full	**Abs**.	Full	**Abs**.	Full
Articles	800	5	150	5	260	4
Words	176146	29583	33827	30305	57256	21791
Proteins	9300	2325	2080	2610	3589	1712

Coreferences	2247	-	463	-	714	-

Relative pronouns	1193	-	254	-	349	-
Pronouns	738	-	149	-	269	-
Definite NPs	296	-	58	-	91	-
Appositions	9	-	1	-	3	-
Others	11	-	1	-	2	-

Events	8615	1695	1795	1455	3193	1294

Gene_expression	1738	527	356	393	722	280
Transcription	576	91	82	76	137	37
Protein_catabolism	110	0	21	2	14	1
Phosphorylation	169	23	47	64	139	50
(with Site)	(67)	(0)	(27)	(12)	(81)	(15)
Localization	265	16	53	14	174	17
(with Loc)	(116)	(12)	(32)	(10)	(111)	(2)
Binding	887	101	249	126	349	153
(with Site)	(138)	(34)	(50)	(114)	(24)	(79)
Regulation	961	152	173	123	292	96
(with Site)	(57)	(8)	(39)	(17)	(11)	(3)
Positive_regulation	2847	538	618	382	987	466
(with Site)	(175)	(7)	(75)	(47)	(37)	(7)
Negative_regulation	1062	247	196	275	379	194
(with Site)	(27)	(9)	(6)	(18)	(10)	(7)

The *events *and the *coreferences *annotations are used for the GE and CO tasks, respectively.

As the full paper collection is a newly added portion for BioNLP-ST 2011, the statistics are examined in more detail across different sections of full papers. As different sections of scientific papers, e.g., title, abstract, introduction, results, conclusions, and so on, are written with different purposes, the type information expected to be found in those sections would be different. Table 3 shows detailed statistics of annotated entities in different sections. For the examination, the sections are roughly classified into *TIAB *(titles and abstracts), *Intro*. (introduction and background), *R/D/C *(results, discussions and conclusions),

**Table 3:** Statistics of annotations in different sections of text

Item	Abstract			Full paper			
			
		All	TIAB	**Intro**.	R/D/C	Methods	Caption
Words	267229	80962	3538	7878	43420	19406	6720

Proteins	14969	6580	336	597	3980	916	751
(Density: P/W)	(5.60%)	(8.13%)	(9.50%)	(7.58%)	(9.17%)	(4.72%)	(11.18%)

Event triggers	11057	3280	216	312	2659	136	173

Events	13603	4436	272	427	3234	198	278
(Density: E/W)	(5.09%)	(5.48%)	(7.69%)	(5.42%)	(7.51%)	(1.02%)	(4.14%)
(Density: E/P)	(90.87%)	(67.42%)	(80.95%)	(71.52%)	(81.93%)	(21.62%)	(37.02%)
(Avg. Coord.: E/T)	(1.23)	(1.27)	(1.26)	(1.37)	(1.23)	*(1.46)*	*(1.61)*

Gene expression	2816	1193	62	98	841	80	112
Transcription	795	204	7	7	140	30	20
Protein catabolism	145	3	0	0	3	0	0
Phosphorylation	355	137	12	12	101	10	2
Localization	492	47	3	15	22	7	0
Binding	1485	380	16	74	266	6	18
Regulation	1426	371	35	30	281	4	21
Positive_regulation	4452	1385	98	131	1087	15	54
Negative_regulation	1637	716	39	60	520	46	51

The *Abstract *column shows the statistics of the abstraction collection (1210 titles and abstracts), and the following columns show that of the full paper collection (14 full papers). *TIAB *= title and abstract, *Intro*. = introduction and background, *R/D/C *= results, discussions, and conclusions, *Methods *= methods, materials, and experimental procedures. Some minor sections, supporting information, supplementary material, and synopsis, are ignored. *Density *= relative density of annotation (P/W = Proteins/Words, E/W = Events/Words, and E/P = Events/Proteins). *Avg. Coord *= average number of coordinated events (E/T = Events/Triggers).

*Methods *(methods and experimental procedures), and *Caption *(captions of figures and tables). An observation at the statistics says that the *Methods *and *Caption *sections mention the events defined in Table 1 much less frequently than the other sections: on average, only one and four events are mentioned in 100 words of *Methods *and *Caption *sections respectively, while 5 ~ 8 events in the other sections. It is also observed that in the two sections, events are mentioned in more coordinated structure: on average, 1.46 and 1.61 events are coordinated in *Methods *and *Caption *sections respectively, while 1.23 ~ 1.37 events are in the other sections. It may agree with the intuition that the two sections usually describe things in a concrete way with much more details than other sections, enumerating relevant entities as exact as possible. Therefor it is expected that the IE from the two sections will benefit from an improved processing of coordinated linguistic structures.

**Figure 3:**
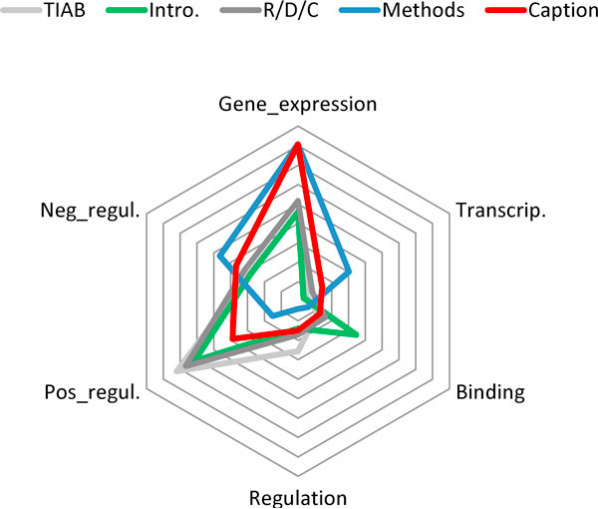
**Event distribution in different sections**. The interval of the contour lines is 5%. For example, in the *Methods *and *Caption *sections, 40% of the events are of *Gene*_*expression*.

The distribution of annotated events across the five different sections is illustrated in Figure 3. It is notable that the *TIAB*, *Intro*. and *R/D/C *sections show similar distribution of annotated events, but the *Methods *and *Caption *sections show significantly different distributions. Particularly, the ratio of Gene_expression is significantly high in the latter two sections, and the ratio of *Negative_regulation *is quite high in the *Methods *section. An intuition which may explain the observation is that the *Methods *sections often describe experimental procedures that are designed to cause negative regulatory effects, e.g., mutation, addition of inhibitor proteins, and so on, and that the results of molecular biology experiments are often observed at the gene expression level. This observation suggests a different event annotation scheme, or a different event extraction strategy would be required for *Methods *and *Caption *sections.

### Task results

The participants to the GE and CO tasks were given three months and seven weeks respectively for system development. After that, 19 teams submitted their final results: 13 to the GE, 4 to the CO, and 2 to both tasks. Table 4 describes the teams who participated in the tasks, except three who wanted to remain anonymous. Table 5 shows brief profiles of the systems. This section presents the final results and analyses on them. The performance is reported in *recall*, *precision*, and *f-score*, based on the *Approximate recursive matching *and the *Protein coreference evaluation *for the GE and CO tasks, respectively. Readers are referred to the Methods section, for more detail.

**Table 4:** Teams who participated in the GE and CO tasks

Team	'09	Task	Background	reference
FAUST	√	12- -	3C	[18]
UMASS	√	12- -	1C	[19]
UTurku	√	123 C	1BI	[20]
MSR-NLP	√	1-- -	4C	[21]
ConcordU		1-3 C	2C	[22]
UWMadison	√	1-- -	2C	[23]
Stanford	√	1-- -	3C+1.5L	[24]
BMI@ASU		12- -	3C	[25]
CCP-BTMG	√	1-- -	3BI	[26]
TM-SCS		1-- -	1C	[27]
XABioNLP		1-- -	4C	[28]
HCMUS		1-- -	6L	[29]
UUtah		--- C	1C	[30]
UZurich		--- C	1C	[31]
USzeged		--- C	2C	-
UCD		--- C	4C	-

The '09 column indicates whether at least one team member participated in BioNLP-ST 2009. In Background column, C=Computer Scientist, BI=Bioinformatician, B=Biologist, L=Linguist

**Table 5:** System profiles

Team		NLP		GE. task		CO. task	
	
	**Lexical Proc**.	**Syntactic Proc**.	**Trig**.	**Arg**.	group	**Mark**.	**Coref**.
FAUST	SnowBall, CNLP	McCCJ+SD	Stacking (UMASS + Stanford)				-
					
UMASS	SnowBall, CNLP	McCCJ+SD	Joint infer., Dual Decomposition				-
					
UTurku	Porter	McCCJ+SD	SVM	SVM	SVM	SVM	SVM
					
MSR-NLP	Porter	McCCJ+SD, Enju	SVM	MaxEnt	rules		-
					
ConcordU	-	McCCJ+SD	dic	rules	rules	rules	rules
					
UWMadison	Morpha, Porter	McCCJ+SD	Joint infer., SEARN				-
					
Stanford	Morpha, CNLP	McCCJ+SD	MaxEnt	MSTParser			-
					
BMI@ASU	Porter, WordNet	Stanford+SD	SVM	SVM	UTurku		-
					
CCP-BTMG	Porter, WordNet	Stanford+SD	Subgraph Isomorphism				-
					
TM-SCS	Stanford	Stanford	dic	rules	rules		-
					
XABioNLP	KAF	-		rules			-
					
HCMUS	OpenNLP	-	dic, rules	rules			-
					
UUtah	GTag	Enju		-		SVM	Reconcile
					
UZurich	LingPipe	Pro3Gres		-		rules	rules
					
USzeged	CTag, Morpha	McCCJ		-		rules	SVM
					
UCD	GTag, LingPipe	-		-		rules	SVM

Proc.=Processing, Trig.=Trigger detection, Arg.=Argument linking, group=Argument grouping, Mark.=Markable detection, Coref.=Coreference linking, SnowBall=SnowBall Stemmer, CNLP=Stanford CoreNLP (tokenization), CTag=CNC Tagger, GTag=Genia Tagger, KAF=Kyoto Annotation Format McCCJ=McClosky-Charniak-Johnson Parser, Stanford=Stanford Parser, SD=Stanford Dependency Conversion.

#### GE Task 1 results

Among the sub-tasks of the GE task, Task 1 was mandatory and 15 teams made their final submissions to the task. Table 6 shows the evaluation results of Task 1. For the evaluation results of the individual simple-type events, e.g. *Gene_expression*, please refer to the GE task overview paper of the BioNLP-ST 2011 workshop [8]. For reference, the reported performance of the two previous systems, UTurku09 and Miwa10 is shown at the top. UTurku09 was the winning system of Task 1 in 2009 shared task [12], and Miwa10 was the best system reported after BioNLP-ST 2009 [2].

**Table 6:** Evaluation results of Task 1 on the (W)hole, (A)bstract, and (F)ull paper collections

Team	Part	Simple Event	Binding	Regulation	All
UTurku09	A	64.21/77.45/70.21	40.06/49.82/44.41	35.63/45.87/40.11	46.73/58.48/51.95

Miwa10	A	70.44	52.62	40.60	48.62/58.96/53.29

	W	68.47/80.25/73.90	44.20/53.71/48.49	38.02/54.94/44.94	49.41/64.75/56.04
FAUST	A	66.16/81.04/72.85	45.53/58.09/51.05	39.38/58.18/46.97	50.00/67.53/57.46
	F	75.58/78.23/76.88	40.97/44.70/42.75	34.99/48.24/40.56	47.92/58.47/52.67
	*F_A_*	66.16/81.04/72.85	45.53/58.09/51.05	39.38/58.18/46.97	50.00/67.53/57.46
	*F_I_*	77.36/71.93/74.55	29.63/36.36/32.65	41.77/50.77/45.83	51.57/56.94/54.13
	*F_R_*	76.34/80.00/78.13	39.00/43.82/41.27	32.66/45.64/38.07	45.98/57.20/50.98
	*F_M_*	48.00/75.00/58.54	*100.0/50.00/66.67*	*20.00/50.00/28.57*	46.88/68.18/55.56

	W	67.01/81.40/73.50	42.97/56.42/48.79	37.52/52.67/43.82	48.49/64.08/55.20
UMass	A	64.21/80.74/71.54	43.52/60.89/50.76	38.78/55.07/45.51	48.74/65.94/56.05
	F	75.58/83.14/79.18	41.67/47.62/44.44	34.72/47.51/40.12	47.84/59.76/53.14
	*F_A_*	64.21/80.74/71.54	43.52/60.89/50.76	38.78/55.07/45.51	48.74/65.94/56.05
	*F_I_*	79.25/82.35/80.77	44.44/48.00/46.15	35.44/56.00/43.41	51.57/65.08/57.54
	*F_R_*	75.95/83.97/79.76	34.00/40.96/37.16	32.29/42.89/36.85	45.09/56.04/49.98
	*F_M_*	48.00/85.71/61.54	*100.0/100.0/100.0*	*20.00/33.33/25.00*	46.88/78.95/58.82

	W	68.22/76.47/72.11	42.97/43.60/43.28	38.72/47.64/42.72	49.56/57.65/53.30
UTurku	A	64.97/76.72/70.36	45.24/50.00/47.50	40.41/49.01/44.30	50.06/59.48/54.37
	F	78.18/75.82/76.98	37.50/31.76/34.39	34.99/44.46/39.16	48.31/53.38/50.72
	*F_A_*	64.97/76.72/70.36	45.24/50.00/47.50	40.41/49.01/44.30	50.06/59.48/54.37
	*F_I_*	84.91/67.16/75.00	25.93/30.43/28.00	30.38/30.77/30.57	47.80/45.24/46.48
	*F_R_*	77.48/78.99/78.23	36.00/30.51/33.03	34.68/45.54/39.38	47.19/54.18/50.44
	*F_M_*	60.00/75.00/66.67	*100.0/20.00/33.33*	*40.00/25.00/30.77*	59.38/50.00/54.29

	W	68.99/74.30/71.54	42.36/40.47/41.39	36.64/44.08/40.02	48.64/54.71/51.50
MSR-NLP	A	65.99/74.71/70.08	43.23/44.51/43.86	37.14/45.38/40.85	48.52/56.47/52.20
	F	78.18/73.24/75.63	40.28/32.77/36.14	35.52/41.34/38.21	48.94/50.77/49.84
	*F_A_*	65.99/74.71/70.08	43.23/44.51/43.86	37.14/45.38/40.85	48.52/56.47/52.20
	*F_I_*	83.02/57.89/68.22	40.74/25.00/30.99	35.44/53.85/42.75	52.20/48.26/50.15
	*F_R_*	78.24/76.49/77.36	37.00/35.24/36.10	35.78/40.21/37.86	48.18/50.93/49.52
	*F_M_*	60.00/75.00/66.67	*100.0/50.00/66.67*	*60.00/50.00/54.55*	62.50/66.67/64.52

	W	59.99/85.53 /70.52	29.33/49.66/36.88	35.72/45.85/40.16	43.55/59.58/50.32
ConcordU	A	56.51/84.56 /67.75	29.97/49.76/37.41	36.24/47.09/40.96	43.09/60.37/50.28
	F	70.65/88.03 /78.39	27.78/49.38/35.56	34.58/43.22/38.42	44.71/57.75/50.40
	*F_A_*	56.51/84.56 /67.75	29.97/49.76/37.41	36.24/47.09/40.96	43.09/60.37/50.28
	*F_I_*	58.49/86.11 /69.66	22.22/50.00/30.77	31.65/40.98/35.71	38.99/56.88/46.27
	*F_R_*	71.37/89.05 /79.24	28.00/53.85/36.84	33.76/44.12/38.25	43.99/58.76/50.32
	*F_M_*	72.00/94.74/81.82	*50.00/20.00/28.57*	*40.00/11.76/18.18*	65.62/51.22/57.53

	W	57.33/71.34/63.57	34.01/44.77/38.66	16.39/25.37/19.91	32.73/45.84/38.19
TM-SCS	A	53.65/71.66/61.36	36.02/49.41/41.67	18.29/27.07/21.83	33.36/47.09/39.06
	F	68.57/70.59/69.57	29.17/35.00/31.82	12.20/21.02/15.44	31.14/42.83/36.06
	*F_A_*	53.65/71.66/61.36	36.02/49.41/41.67	18.29/27.07/21.83	33.36/47.09/39.06
	*F_I_*	71.70/67.86/69.72	18.52/31.25/23.26	12.66/27.78/17.39	33.33/49.07/39.70
	*F_R_*	66.03/69.76/67.84	32.00/37.65/34.59	11.38/19.68/14.42	29.44/41.20/34.34
	*F_M_*	72.00/72.00/72.00	*50.00/50.00/50.00*	*20.00/12.50/15.38*	62.50/57.14/59.70

The full paper collection is further classified to titles/abstracts (*F_A_*), introductions (*F_I_*), results/dicussions/conclusions (*F_R_*), and methods (*F_M_*). Evaluated performance is reported in recall/precision/f-score. Some notable figures are underlined.

The best overall performance on Task 1 (56.04%) in BioNLP-ST 2011 was achieved by the FAUST system, which adopted a combination model of UMass and Stanford. In terms of improvement, the performance of FAUST on the *abstract collection *(57.46%) demonstrates a significant improvement of the community on the GE task, when compared to the performance of UTurku09 (51.95%) and Miwa10 (53.29%). The biggest improvement was made to the Regulation events (from 40.11% and 40.60% to 46.97%) of which the extraction requires a complex modeling of recursive event structure - an event may become an argument of another event. In terms of generalization, the performance of UMass on the *full paper collection *(53.14%) suggests that the technology which began with only abstracts can be generalized to full papers without a big loss of accuracy. Note that however this observation contrasts to the recent report about a substantial performance drop of protein mention detection in full papers [13], and that the performance reported in this paper is obtained when the gold protein annotation is given. Therefore, the performance of event extraction in a full automatic system needs to be investigated and discussed more carefully.

The ConcordU system is notable as it is the sole rule-based system that is ranked above the average. The performance of the system demonstrates both pros and cons of a typical rule-based approach. It showed the best precision in extracting simple-type events, but was not very successful with complex-type events, suggesting that when the problem is simple, rules may be developed effectively, but it may become difficult as the problem gets complex. The performance of the system on the two collections shows that the rules of ConcordU system well generalize to full papers.

The generalization of performance can be further investigated by observing the performance on different sections. As noted in the previous section, the *Methods *sections of full papers are significantly different from other sections, while the *Intro*. and *R/D/C *as well as *TIAB *are relatively similar to the *abstract collection*. It is thus expected that generalization to the *Methods *sections be more difficult than to other sections.

At a glance, the overall performance reported in Table 6 does not seem worse on the *Methods *sections than on other sections. However, it needs to be considered that the *Methods *sections do not include as many complex type events as other sections. In fact, in the *Methods *sections of the test data set, the numbers of *Binding *and *Regulation *events are both less than 10, which prevents a reliable analysis and that is the reason that the performance figures are italicized in the table. In the *Methods *sections, simple type events take almost 80% of the whole event, thus the overall performance is dominated by the performance extracting simple-type events. In such a case, it is more reasonable to refer to the simple event performance than to the overall performance. The simple event performance reported in Table 6 supports the hypothesis that the performance be harder to generalize to the *Methods *sections.

The two rule-based systems, ConcordU and TM-SCS, however seem free from the hypothesis, showing the best performances (81.82% and 72.00%) on the *Methods *sections. We find the reason of their good performance on the sections from the fact that rule-based approaches are in general not aggressive as much as machine learning approaches in optimizing to the training data. Note that there is usually a trade-off between optimization and generalization.

This time, three teams achieved better results than Miwa10, which indicates some role of focused efforts like BioNLP-ST. The comparison between the performance on abstract and full paper collections shows that generalization to full papers is feasible with very modest loss in performance.

#### GE Task 2 results

Task 2 of the GE task was optional and 4 teams submitted their final results. Table 7 shows the final evaluation results. For reference, the reported performance of the task-winning system in 2009, UT+DBCLS09 [14], is shown at the top. The top two systems on Task 1, FAUST and UMass, marked the best performances on Task 2, too. The performances of the two systems on the *abstract collection *demonstrate a significant improvement of the technology in two years (from 44.52% to 52.77% and 52.12%).

**Table 7:** Evaluation results of Task 2 on the (W)hole, (A)bstract, and (F)ull paper collections

Team		Sites (222)	Locations (66)	All (288)
UT+DBCLS09	A		23.08/88.24/36.59	32.14/72.41/44.52

	W	32.88/70.87/44.92	36.36/75.00/48.98	33.68/71.85/45.86
FAUST	A	43.51/71.25/54.03	36.92/77.42/50.00	41.33/72.97/52.77
	F	17.58/69.57/28.07	-	17.39/66.67/27.59

	W	31.98/71.00/44.10	36.36/77.42/49.48	32.99/72.52/45.35
UMass	A	42.75/70.00/53.08	36.92/77.42/50.00	40.82/72.07/52.12
	F	16.48/75.00/27.03	-	16.30/75.00/26.79

	W	32.88/62.93/43.20	22.73/83.33/35.71	30.56/65.67/41.71
BMI@ASU	A	37.40/67.12/48.04	23.08/83.33/36.14	32.65/70.33/44.60
	F	26.37/55.81/35.82	-	26.09/55.81/35.56

	W	40.09/65.44/49.72	00.00/00.00/00.00	30.90/65.44/41.98
UTurku	A	48.09/69.23/56.76	00.00/00.00/00.00	32.14/69.23/43.90
	F	28.57/57.78/38.24	-	28.26/57.78/37.96

Evaluated performance is reported in recall/precision/f-score. Some notable figures are underlined.

In detail, a significant improvement was made for *Location *arguments (36.59%→50.00%) by the top two systems. The performance of *site *argument extraction was also improved significantly. A breakdown of the evaluation results of *site *argument extraction, shown in table 8, indicates that for *Phosphorylation *events the performance of finding the *site *arguments is approaching a level of practical use (82.93% by UTurku). As for *Regulation*, the performance was significantly improved (41.03% by FAUST), while the performance improvement for *Binding *was only marginal (13.33% by BMI@ASU).

**Table 8:** Evaluation results of Site extraction for different event types

Team		Phospho. (67)	Binding (84)	Reg. (71)
UT+DBCLS09	A	71.43/71.43/71.43	04.76/50.00/08.70	12.96/58.33/21.21

	W	71.64/84.21/77.42	05.95/38.46/10.31	28.17/60.61/38.46
FAUST	A	71.43/81.63/76.19	04.76/14.29/07.14	29.63/66.67/41.03
	F	72.73/100.0/84.21	06.35/66.67/11.59	23.53/44.44/30.77

	W	76.12/79.69/77.86	04.76/36.36/08.42	22.54/64.00/33.33
UMass	A	76.79/76.79/76.79	04.76/14.29/07.14	22.22/70.59/33.80
	F	72.73/100.0/84.21	04.76/75.00/08.96	23.53/50.00/32.00

	W	52.24/97.22/67.96	20.24/53.12/29.31	29.58/43.75/35.29
BMI@ASU	A	53.57/96.77/68.97	09.52/22.22/13.33	31.48/51.52/39.08
	F	45.45/100.0/62.50	23.81/65.22/34.88	23.53/26.67/25.00

	W	76.12/91.07/82.93	21.43/51.43/30.25	28.17/44.44/34.48
UTurku	A	78.57/89.80/83.81	09.52/18.18/12.50	31.48/54.84/40.00
	F	63.64/100.0/77.78	25.40/66.67/36.78	17.65/21.43/19.35

Evaluated performance is reported in recall/precision/f-score. Some notable figures are underlined.

The performance on the *full paper collection *shown in Table 7 seems to tell the extraction of secondary arguments in full text is much more challenging, but Table 8 shows there is no particular performance degradation on the *full paper collection*, except with the *Regulation *events. The reason of the the low overall performance figures on the *full paper collection *in Table 7 is explained by referencing Table 2. In the full paper portion of the test set, there were extraordinary number of *Binding *events with *site *arguments (70%), which dominated the overall performance figures. Note that it cancels the conclusion made by [10] that extraction of secondary arguments from full papers might be much more challenging than from abstracts.

#### GE Task 3 results

Table 9 shows final evaluation results of Task 3. For reference, the reported performance of the task-winning system in 2009, ConcordU09 [15], is shown at the top. Among the two teams participated in the task, UTurku showed a better performance in extracting negated events, while ConcordU showed a better performance in extracting speculated events. Both demonstrate significant improvements over the ConcordU09 system, but the performances are still far from a level of practical use. It seems generalization to the full papers is a bit more challenging with the speculation extraction.

**Table 9:** Evaluation results of Task 3 on the (W)hole, (A)bstract, and (F)ull paper collections

Team		Negation	Speculation	All
ConcordU09	A	14.98/50.75/23.13	16.83/50.72/25.27	15.86/50.74/24.17

	W	22.87/48.85/31.15	17.86/32.54/23.06	20.30/39.67/26.86
UTurku	A	22.03/49.02/30.40	19.23/38.46/25.64	20.69/43.69/28.08
	F	25.76/48.28/33.59	15.00/23.08/18.18	19.28/30.85/23.73

	W	18.77/44.26/26.36	21.10/38.46/27.25	19.97/40.89/26.83
ConcordU	A	18.06/46.59/26.03	23.08/40.00/29.27	20.46/42.79/27.68
	F	21.21/38.24/27.29	17.00/34.69/22.82	18.67/36.14/24.63

Evaluated performance is reported in recall/precision/f-score. Some notable figures are underlined.

#### CO task results

Table 10 shows the evaluated performance of the six systems who participated in the CO task. The UUtah team, who already had an experience of developing a coreference resolution system for the newswire domain, marked the best performance (34.05%). The authors reported a performance degradation (from 66.38% to 49.64% in MUC score) by the change of the domain. For a more detailed analysis, the performance was evaluated for different types of anaphoric expressions. As shown in the table, the three top-ranked teams, UUtah, UZurich and ConcordU, marked the best performances in finding coreferences of definite noun phrases (10.8%), pronouns (26.7%) and relative pronouns (66.2%), respectively. It is not surprising that the coreference resolution for relative pronouns marked the highest accuracy as in many cases relative pronouns immediately follow their antecedents. Coreference resolution of definite noun phrases marked very low accuracy even by the top-ranked system. The reason may be found in the fact that most systems relied on syntactic features, e.g., part-of-speech or syntactic parse, for coreference resolution without differentiating the type of coreferences. However, in many cases there is only semantic connection between a definite noun phrase and its antecedent. The UUtah system does incorporate some semantic features which are originally designed for newswire domain, but it was once reported that semantic features are not domain portable while syntactic features are [16]. So, it seems there is much room for improvement especially for definite noun phrases by developing effective semantic features for the biomedical domain.

**Table 10:** Evaluation results of the CO task

Team	Relative pronoun	Pronoun	DNP	All
UUtah	56.0/71.2/62.7	12.0/79.0/20.8	05.5/66.7/10.1	22.2/73.3/34.1
UZurich	46.7/71.4/56.5	17.9/62.9/27.5	04.1/12.5/06.2	21.5/55.5/31.0
ConcordU	68.0/64.6/66.2	-	-	19.4/63.2/29.7
UTurku	29.3/73.3/41.9	12.8/72.7/21.8	01.4/14.3/02.5	14.4/67.2/23.8
USzeged	-	-	-	03.2/03.5/03.3
UCD	-	-	-	00.7/00.3/00.4

Evaluated performance is reported in recall/precision/f-score. *DNP *= definite noun phrase. Some notable figures are underlined.

## Conclusions

The Genia event task which was repeatedly arranged for BioNLP-ST 2009 and 2011 took a role of measuring the progress of the community and generalization of IE technology to full papers. The results from 15 teams who made their final submissions to the task show a clear progress of the community in terms of the performance on a focused domain and also generalization to full papers.

The coreference resolution supporting task of BioNLP Shared Task 2011 has drawn attention from researchers of different interests. Although the overall results are not good enough to be helpful for the main shared tasks as expected, the analysis results show the problems to be solved and directions for improvements.

## Methods

### GE task evaluation

The shared task requires participants to predict event annotation for the test data. The evaluation is carried out by comparing the predicted annotation to the gold annotation. For the comparison, equality of annotations is first defined at various levels as follows:

(1) Event equality

holds between any two events when

(1-1) the event types are the same,

(1-2) the event triggers are the same, and

(1-3) the arguments are fully matched.

(2) Argument equality

holds between any two arguments when

(2-1) the role types are the same, and

 (2-2-1) both are text entities in equality, or

 (2-2-2) both are events in equality.

(3) Text entity equality

holds between any two text entities when

(3-1) the entity types are the same, and

(3-2) the spans are the same.

In the condition (1-3), a full matching of arguments between two events means there is a perfect one-to-one mapping between the two sets of argument, while the equality of individual arguments is defined by the Argument Equality. Due to the condition (2-2-2), event equality is defined recursively for events referring to events. Any two text spans (*beg*1, *end*1) and (*beg*2, *end*2), are the same iff *beg*1 = *beg*2 and *end*1 = *end*2. Note that the event triggers are also text entities thus their equality is defined by the text entity equality. Various evaluation modes can be defined by varying the condition of equality. In the following, we describe five fundamental variants applied in the evaluation.

#### Strict matching

The *strict matching *mode requires exact equality, as defined in previous section. As some of its requirements may be viewed as unnecessarily precise, practically motivated relaxed variants, described in the following, are also applied.

#### Approximate span matching

The *approximate span matching *mode is defined by relaxing the requirement for text span matching for text entities. Specifically, a given span is equivalent to a gold span if it is entirely contained within an extension of the gold span by one word both to the left and to the right, that is, *beg*1 ≥ *ebeg*2 and *end*1 ≤ *eend*2, where (*beg*1, *end*1) is the given span and (*ebeg*2, *eend*2) is the extended gold span.

#### Approximate recursive matching

In strict matching, for a regulation event to be correct, the events it refers to as theme or cause must also be be strictly correct. The *approximate recursive matching *mode is defined by relaxing the requirement for recursive event matching, so that an event can match even if the events it refers to are only partially correct. Specifically, for partial matching, only Theme arguments are considered: events can match even if referred events differ in non-Theme arguments.

#### Event decomposition mode

Many events are expressed with more than one argument, e.g., binding of multiple proteins or regulation with a theme and a cause. Such events are inherently more difficult to extract than events with a single argument. In the *Event decomposion mode*, events with multiple arguments are decomposed into multiple single-argument events. Specifically, in this mode, each multi-argument event

trigger, arg1-type:arg1-value, arg2-type:arg2-value, . . .

is decomposed into single-argument events

trigger, arg1-type:arg1-value

trigger, arg2-type:arg2-value

. . .

The resulting single-argument events are treated as separate events in evaluation, thus allowing recognition of partially correct events and awarding the recognition of complex events more highly. Note that the *Event decomposition mode *is used in combination with other matching modes.

### CO task evaluation

The coreference resolution performance is evaluated in two modes.

The *Surface coreference mode *evaluates the performance of finding anaphoric protein references and their antecedents, regardless whether the antecedents actually embed protein names or not. In other words, it evaluates the ability to predict the coreference relations as provided in the gold coreference annotation file, which we call *surface coreference links*.

The *protein coreference mode *evaluates the performance of finding anaphoric protein references with their links to actual protein names (*protein coreference links*). In the implementation of the evaluation, the chain of surface coreference links is traced until an antecedent embedding a protein name is found. If a protein-name-embedding antecedent is connected to an anaphora through only one surface link, we call the antecedent a *direct protein antecedent*. If a protein-name-embedding antecedent is connected to an anaphora through more than one surface link, we call it an *indirect protein antecedent*, and the antecedents in the middle of the chain *intermediate antecedents*. The performance evaluated in this mode may be directly connected to the potential performance in main IE tasks: the more the (anaphoric) protein references are found, the more the protein-related events may be found. For this reason, the protein coreference mode is chosen as the primary evaluation mode.

Evaluation results for both evaluation modes are given in standard recall, precision and f-score.

#### Surface coreference

*Surface coreference links *are links between target anaphors and their antecedents or intermediate antecedents. Note that the shared task development and training data include not only the target protein coreference links but also other pronoun and definite noun phrase coreference links.

A response expression is matched with a gold one following partial match criterion. In particular, a response expression is considered correct when it covers the minimal boundary, and is included in the maximal boundary of expression. Note that maximal boundary is the span of expression annotation, and minimal boundary is the head of expression, as defined in MUC annotation schemes [17]. A response link is correct when its two argument expressions are correctly matched with those of a gold link.

#### Protein coreference

This is the primary evaluation perspective of the protein coreference task. In this mode, we ignore coreference links that do not reference to proteins. Intermediate antecedents are also ignored.

Protein coreference links are generated from the surface coreference links. A protein coreference link is composed of an anaphoric expression and a protein reference that appears in its direct or indirect antecedent. Below is an example.

Example:

R1 Coref Ana:T29 Ant:T28 [T5, T4]

R2 Coref Ana:T30 Ant:T27

R3 Coref Ana:T32 Ant:T31 [T10]

R4 Coref Ana:T33 Ant:T32

In this example, supposing that there are four surface links in the coreference annotation file (T29,T28), (T30,T27), (T32,T31), and (T33, T32), in which T28 contains two protein mentions T5, T4, and T31 contains one protein mention T10; thus, the protein coreference links generated from these surface links are (T29,T4), (T29,T5), (T32,T10), and (T33, T10). Note that that T33 is connected with T10 through the intermediate expression T32.

Response expressions and generated response result links are matched with gold expressions and links correspondingly in a way similar to the surface coreference evaluation mode.

## Competing interests

The authors declare that they have no competing interests.

## Authors' contributions

JD conceived the Genia and Protein Coreference tasks and prepared the benchmark data sets. JD and YW implemented the Genia task evaluation and analyzed the results. NN implemented the Protein Coreference task evaluation and analyzed the results. JD and NN prepared the manuscript. JT, TT and AY participated in the discussions and finalization of the manuscript.
